# Bibliographical Notices

**Published:** 1855-04

**Authors:** 


### BIBLIOGRAPHICAL NOTICES.

*On the topical Medication of the Larynx in certain Diseases of the Respiratory and Vocal Organs.* By EBEN. WATSON, A.M., M.D., &c. London: 1854. 8vo. pp. 183.

*On the Employment of Injections into the Bronchial Tubes, and into Tubercular Cavities of the Lungs.* By HORACE GREEN, M.D., LL.D. [" The American Medical Monthly," Jan. 1855.]

The topical medication of the larynx which would seem to have originated with Sir Charles Bell in 1816, and with M. Bretonneau in 1818, was, as every body knows, first popularized by MM. Trousseau and Belloc in 1836. A translation of the Essay by the last named gentlemen was published in this country in 1841, and five years afterwards Dr. Horace Green put forth his " Treatise on Diseases of the Air Passages." This writer has succeeded in attracting a good deal of attention from the public and the profession, although neither his method nor his pretensions have met with general acceptance with the latter. This must be mainly attributed to the uncandid statements and unscientific descriptions contained in his essays, by which many who would have been tempted to put the method into practice, were deterred from examining it, and inspired with a disrelish for the whole matter.

It was at first doubted whether the probang used by Bretonneau could be passed beyond the vocal chords, and although the greater number of persons no longer deny the possibility of this manœuvre, some are still incredulous, among whom no less names may be mentioned than Erichsen, who is convinced " that the sponge has never been passed, in the living subject, beyond the true vocal chords," and Trousseau who is stated to be equally unbelieving. But Dr. Green has resorted to a very ingenious expedient to put these gentlemen in the wrong. It seems that Dr. Marshall Hall, on his late visit to the United States, was still of the number of those who believed that the probang passed into the œsophagus and not into the trachea, and he suggested as an *experimentum crucis* that the tube introduced should consist of a catheter through which the air could freely pass. Subsequently this was done, and the patient breathed through the artificial trachea so freely as to extinguish a candle placed at its further end, even when the lips and nose were so far closed as to prevent the discharge of any air from them; besides which an India rubber bag tied over the open end of the tube was seen alternately to collapse and expand as the patient breathed. These conclusive experiments are said to have been witnessed by several physicians of well known skill and reputation for veracity.

Whatever doubts may have been, or still may be entertained regarding the possibility of introducing a probang into the larynx, an experiment such as that just described having once been performed must put an end to them, however much people who are accustomed to attach considerable importance to respiration as a means of "getting one's living," may shake their heads, and suggest the possibility of its being after all no more than a conjurer's trick. Just as Dr. Watson, with all his knowledge and skill, finds his belief staggered by certain other statements of his American confrère. It seems that Dr. Green wrote as follows to the *Lancet*:—"In cases where I have had reason to believe that ulcerations of the membrane had extended below the bifurcation, I have employed a probang nearly straight, and have pushed this instrument not only to the division of the trachea, but *turning it aside*, have passed it at will, *in many instances into the right or left bronchus*, with as much ease and safety as the catheter is introduced into the bladder." After pointing out that such a statement as this is not likely to convince the incredulous, and characterizing the case which Dr. Green adduces to support it, "as more objectionable than the statement it was intended to corroborate," Dr. Watson says, "Of those who can admit the possibility of the treatment to which the patient was subjected, few will venture to assent to the diagnosis of the ulcer in the right bronchus, and still fewer will affirm that the aphonia could be occasioned by such an ulcer, supposing it to have existed, or was likely to be removed on its cure."

Nor is Dr. Watson quite able to digest the sponge, which, according to Dr. Green, dropped into the windpipe of a man whose larynx Dr. Peaslee was cauterizing. The statement, he fears, has done "much damage to the cause which Dr. Green adduced it to support; for by the most undoubted rule of arithmetic, the worthy professor would have us believe that the sponge he removed from the trachea (or right bronchus, for it is not very clear which is meant) was one half inch multiplied by fourteen in diameter, viz., seven inches ; and, indeed, that the whole mass was even larger than this at first; that it was seven and a half or eight inches, i. e., two thirds of a foot in diameter. Now this is simply ridiculous, &c."

It appears, too, that while Dr. Green "recommends the use of a bent spatula, with which he seems able to expose the parts to view;" and while Dr. A. C. Post has, "in a number of instances, exposed distinctly to view the laryngeal surface of the epiglottis," the less fortunate Dr. Watson has been "unable to see any part of the larynx, except, in certain cases, the *tip* of the epiglottis." (p. 69.) Dr. W. also rejects the use of a bent spatula, and assures us that "the only way of assuring one's self that he enters the rima-glottidis, is to introduce the fore finger of the left hand into the mouth of the patient, passing it over the root of the tongue, till its point comes in contact with the top of the epiglottis. By now maintaining the finger in this position, and cleverly passing the sponge of the laryngeal probang over it, the rima-glottidis is reached with perfect certainty." (p. 23.) The introduction of a sponge through this opening he admits to be opposed by the actual narrowness of the passage, as well as by the spasmodic closure of the glottis, and he therefore insists upon the necessity of educating the part to bear this manœuvre, and seizing a moment of inspiration to accomplish it. It will be seen, therefore, that the operation is not quite so simple as it has pleased some persons to say that it is.

In his chapter on the mode of action of topical agents used in laryngeal disease, Dr. Watson is hardly more successful than other writers upon the phenomena and cure of inflammation. In one place (p. 33) he says that when a caustic is applied to the inflamed web of a frog's foot, "the *stimulant* solution causes a renewed and increased *dilatation* of the blood vessels, and the retarded current moves on in them more freely than before," while (p. 88) it is afterwards stated that the topical application "removes the remnant of inflammatory action which had still lingered in the glottis, by causing *contraction* of its blood vessels." These statements we are altogether unable to reconcile with one another ; each seems to have been suggested by the discussion on which the author was more immediately engaged, and neither is applicable to all the results of the topical use of the caustic solution.

In several successive chapters the topical treatment is described in its application to acute and chronic laryngitis, aphonia, hooping cough, spasmodic asthma and spasmodic coughs, laryngismus, and the laryngeal complications of pulmonary phthisis.

In simple acute laryngitis the author warns us not to use cauterization until after the inflammatory symptoms are subdued, nor to employ a strong solution when these have been very active. In membranous croup he is altogether opposed to its employment, unless before any exudation has been poured out, (p. 50) which is equivalent to saying before there is any croup to treat. He analyses the cases which Dr. Green published as cases of "croup" treated by caustic, and comes to the conclusion, long ago demonstrated in this country, that in only six out of the thirteen cases the symptoms resemble those of true croup, and that there is nothing to show any favorable result in these. Indeed Dr. Watson has found in cases of croup "the symptoms of congestion in the laryngeal lining, such as pain and difficulty of breathing, increased by the application of even a weak solution of nitrate of silver, and the very act of applying the solution is hurtful in these cases." The large and intelligent experience of our author is, it will be observed, directly opposed not only to that of Dr. Green, but also to the results of so candid and enlightened an observer as Dr. Ware.

As regards "follicular disease of the larynx," a stalking-horse from behind which Dr. Green has acquired so large a *clientelle*, Dr. Watson is disposed to regard it altogether as what Carlyle would call "a sham." To this purpose he cites, as others have done before, the results of pathological investigations by Louis, Trousseau, Belloc, Porter, and Copland, and declares that there is no evidence, even in Dr. Green's writings, "which is worthy to be considered as establishing the matter," and that "the assumption of the existence of such a lesion as the cause of certain symptoms is neither necessary nor philosophical." (p. 67.) He, on the contrary, connects follicular pharyngitis with dyspeptic derangements just as Dr. Chapman did originally, and insists that whatever else the practitioner does, "these must be set right before his patient is cured."

Aphonia is referred by Dr. Watson to lesions affecting the glottis, such as the thickening or the palsy of its muscles, and to others consisting of relaxation, thickening, or *ulceration* of the mucous membrane above or below the glottis. Of the last mentioned lesion which has been so largely speculated upon as a trading capital, and the existence of which in chronic disease, independently of syphilis or tuberculosis, has generally been denied by pathological writers, Dr. Watson remarks that he believes in their existence "independently of tubercle in the lung, in a class of cases which, however, are neither numerous nor ever quite free from the possibility of doubt as to the diagnosis."

The chapter on the laryngeal treatment of hooping cough is extremely interesting. The author believes that if the local and inflammatory element is met by cauterization after the acute stage is passed, the spasmodic element will of itself subside. As to the practical results they are indeed most encouraging. Taking the cases treated by M. Joubert and by Dr. Watson, we find that 57.4 per cent. were cured in two weeks, and of the remainder 36.5 per cent. were cured within four weeks. The usual treatment, on the other hand, requires an average duration of one and a half to three and a half months. The ordinary strength of the solution employed by Dr. Watson is of fifteen grains to the ounce, but weaker at first than subsequently when the inflammation has subsided. He points out as conditions of success the regulation of the patient's digestion so as to prevent vomiting, the use of the caustic every day or every second day when the stomach is empty, the use of the finger or the handle of a teaspoon instead the "formidable spatula," and the avoiding to enter the larynx while it is still in a state of catarrhal inflammation.

The usefulness of cauterizing the larynx in spasmodic asthma is maintained by Dr. Watson. He very properly excludes from consideration cases of organic disease, such as emphysema and organic affections of the heart, but he assumes that closure of the glottis is an important element of the nervous disease, with which also spasm of the trachea and of the ultimate bronchia combine. It cannot be claimed that any of the cases adduced or of the arguments employed in the least degree demonstrate either the statement just made or the efficacy of the proposed treatment in spasmodic asthma. All of the cases involve an inflammatory element, and the nervous element is far from constituting the whole disease. It is therefore useless, we think, to examine the influence of laryngeal cauterization upon them.

The caustic is very properly regarded as of secondary importance in those cases of cough which are said to depend upon dyspepsia, but which are in reality connected with the deranged state of the muciparous follicles of the pharynx which complicates that of the stomach. Attention to the dyspeptic symptoms will here effect a cure even without the topical medication, but this latter hastens and confirms it. A similar remark will apply to hysterical coughs; but in neither case is the caustic essential, or capable of performing what other remedies cannot.

In the chapter on Laryngismus the author considers this symptom chiefly as one of the phenomena of epilepsy, and claims that cauterization of the larynx mitigates the spasms by correcting this element of the attack. But all cases of epilepsy are not marked by laryngismus, and hence all are not suited for this operation any more than for that of tracheotomy proposed by Dr. Marshall Hall.

The Laryngeal Complications of Pulmonary Phthisis are treated of in the last chapter of the work before us. The author endeavors to prove that the cough is not only an annoyance to the patient, but a serious aggravation of the disease by occasioning hæmoptysis, and increasing the pulmonary affection. It is to be feared that his ingenious argument amounts to nothing more than an attempt to make out a case; for, as every one knows, hæmoptysis is apt to be one of the earliest symptoms of consumption, and often occurs before any cough whatever exists, while the cases in which violent coughing produces it are exceedingly rare. When the larynx itself is affected by inflammation or by ulcers, undoubtedly these conditions are aggravated by the cough, and their cure, if that can be accomplished, or their quiescence, if it cannot, is to be sought by every possible expedient as a means of promoting the patient's comfort, and even of prolonging his life. The topical treatment of the larynx will sometimes secure this end; but the author cautions us against its use, in very acute cases and during acute exacerbations of chronic cases. The illustrations which he publishes show nothing more than the palliation of the accidental laryngeal symptoms, and he carefully avoids asserting that he has effected a cure of consumption in any case whatever. In this he is truthful, and more modest than his American prototype, who was wont to mystify his readers by an ingenious play upon the word *tuberculous*, which meant enlarged follicles or genuine tubercles, according to the exigencies of the occasion.

In the paper of Dr. Green we find that he employed his ability to pass the probang into either bronchus, for the purpose of applying a solution of nitrate of silver to the diseased membrane of the bronchi, and even of injecting, "if possible," the same solution into tubercular excavations. His first case presented "a large cavity in the apex of the left lung." He introduced "an elastic tube thirteen inches long through the trachea and into the left bronchial division," and through it "injected one drachm of a solution of nitrate of silver, of the strength of forty grains to the ounce of water into the lung." A few minutes afterwards "the patient stated that she felt a warm sensation in the upper portion of the left lung," i. e., in the seat of the cavity. Apart from the bold assertion that the instrument was introduced into the left bronchus, we are called upon to believe that a drachm of liquid, ascended against the force of gravity from the root to the apex of the lung, and that it produced a warm sensation in a cavity which could possess no sensibility whatever, instead of being felt in the lower bronchia where alone it could have been diffused. On reading over these details once more we cannot but ask, are they meant seriously? or is the author, having already tested the credulousness of so many physicians, amusing himself quietly at their expense?

After this first *successful* experiment, Dr. Green states that he treated thirty-two patients laboring under tubercular or bronchial diseases by the same method; and nine of them presented cavities in the lungs. All of them, it is stated, appeared to be benefitted, and some of them greatly, and "in those cases where tubercles exist, whether the exudation be in a crude state, or beginning to soften, the beneficial effects of the treatment have been thus far as uniform and certain, although the improvement has not been as rapid in these as in the former cases, [viz., of chronic bronchitis.] Most of these cases are still under treatment, and the final result cannot be foretold." On the contrary, we think that nothing is easier. Meanwhile, let our readers observe out of what stuff medical experience may be manufactured.

The following passage from Dr. Watson's work (p. 28) will serve as a commentary upon the subject of the last paragraph, which we cannot trust our pen to write of as it deserves.

"I need only now remark, in conclusion, that after the probang has passed through the glottis, there is no obstacle to prevent its being carried down the whole length of the trachea; but this must be done so hurriedly that no deliberate turning of the instrument into either division of the bronchus can in my opinion be accomplished; and I know from experience that few or none will be found so calm and so self-possessed as to submit to its being even attempted. Whenever the patient feels that the *besoin de respirer* is becoming strong, all other thoughts are dismissed, and he struggles to rid himself of the obstructing cause. The passage of the probang, even quickly, down part of the trachea, is itself a severe operation, and should never be performed upon a patient who is not habituated to the application of the remedy to the upper parts of the windpipe."

---

*Autobiography of Charles Caldwell M. D, with a preface, notes, and appendix*, by HARRIET W. WARNER. Philadelphia: Lippincott, Grambo, & Co. 1855. One vol. Oct. pages 454.

To review these 454 pages to our own satisfaction, would require more space than can be awarded to them in a medical journal; we shall therefore do little more than notice some points that cannot fail to interest the medical public. The book is deeply fraught with a mixture of good and evil, either of which it is well adapted to teach, according to the mental habits of the reader. But as all the good it contains is amply supplied by other and better books, we cannot fail to wish, that the distinguished author had given it, as Petrarch said of his letters, *to Vulcan to correct.*

Autobiography is a very difficult and dangerous task, owing to the malevolent passions that may be enlisted in the work. Selfishness and vanity lead an author to overestimate himself and to depreciate others; malice and revenge are ever ready to play their hateful part; and a long life of envy must cloud the mind of the octogenarian writer with vapors of poison.

From all these embarrassing influences the two autobiographers, Franklin and Priestly, whom Dr. C. often refers to as noble exemplars, are totally free. Their success in life far transcended their hopes, and finally placed them on that shining eminence from which they looked back on life as on a cloudless day, wherein nothing was seen that could disturb their serene satisfaction. All the noxious vapors which had sometimes annoyed them in their long journey of life, were now lost in one cloudless glory; and they went down the hill of life plucking flowers and fruits, as also reveling in sweet recollections.

Such was not the happy lot of Dr. C. He had, by his own confession, fixed his ambitious heart on a certain chair in the University of Pennsylvania, and when this had become vacant by the death of Rush, he had the mortification to see it filled by one who was not only his personal enemy but was also greatly inferior to himself. Yet a harder fate than even this awaited him; for this hated incumbent soon passed away, and our longing aspirant was again set aside by the "superior tact" of his particular friend, whom he tho't far less qualified than himself in the various attributes of a professor of medicine.

Thus doubly mortified he soon determined to leave Philadelphia, where he had laboured hard for 27 years, in expressly preparing himself for a teacher in the great American school and in the very chair long honoured by the eloquence of Rush.

To suppose that the presumed causes of this cruel disappointment did not rankle in his mind, is to demand for the author more Christian charity than many will accord to him or even claim for themselves. He says plainly, that he considered Rush as the principal cause of his failure, and hence he has written much of that great and good man that cannot be received but as reveries often repeated, till they are believed to be facts. That his memory had greatly failed, I know from his incorrect relation of one fact at which I was present—the hissing scene related at p. 294. He was at the upper part of the chemical theatre and when the lecture was closed and the students were rushing away, a few of them called out *Dr. Caldwell* and hissed. He received this with apparent placidity, I suppose contemptuous, and then striding over the benches, he said to the professor very pleasantly —"no animals hiss except snakes and geese."

But his deceitful reveries made other impressions on his mind and he says—

"I then advanced into a more conspicuous part of the room, and with a menacing action of my arms toward the place from which the sound had reached me, exclaimed in a calm and contemptuous voice: 'I know of but three sorts of vermin that vent their spleen by hissing; an enraged cat, a viper, and a goose; and I knew not till now, that either of them infested this room.' On this, from the same quarter came the cry: 'Turn him out! turn him out!' And there was immediately around me a party of my own pupils, chiefly from the States of Georgia and Kentucky, to whom I was communicating instruction by lectures and examinations; and who, apprehensive that I might be assaulted, requested me to accompany them out of the room, and they would protect me. My immediate reply, calm and courteous, but as positive as words and manner could make it, was: 'I thank you gentlemen, for your proffered kindness; but I do not need it. I can protect myself.' Raising then my voice, so as to be heard throughout the room, I added: 'From this spot I will not move, until those insolent fellows shall have left the room, unless they remain in it (looking at my watch) until twelve o'clock, at which time I must leave it to make good an engagement. And should any one of them have the audacity to approach me as an assailant, he shall have abundant cause to remember his imprudence and deplore his rashness until the end of his life, which may perhaps be nearer at hand than they are prepared to imagine, for I will precipitate him to the bottom of this pit, and determine by experiment which is the thicker and harder, his brain-pan or that brick floor."

Not one word of this speech was made, nor was there a word said about *turning him out.* He has related what he was accustomed to talk to himself in his moments of wrath, and probably to dream over at night, till the truth was at last supplanted and prolific error deeply rooted. In the real scene, the *angry cat* was not named; but in after times, she was very happily brought in, to make the speech accord with his increase of knowledge.

It must have been this forty-five years revery on old irritations, that led him to make some stories concerning Dr. Rush that cannot be received. No man in his right mind can credit his relation of a scene at his graduation; no one can for a moment believe that a young student stood forth in the presence of trustees, professors and "many of the most distinguished members of the Bench and Bar, the Pulpit and the citizens of Philadelphia at large, together with most of the respectable strangers in the city," see p. 236 :—I say it is not credible that a student could have stood before this auditory "drawn up to his full height with folded arms, looking slowly and significantly," making the contentious speeches which his octogenarian pen relates; nor will any man who knew Dr. Rush believe the part attributed to him. He was a man of uncommon dignity of deportment and elegance of manners, one who had long associated with the best exemplars, had been no little accustomed to collisions of intellect, and was therefore not easily driven from his propriety by an impudent student, though *drawn up, as he says, to his full height*—nearly 6½ feet—*with folded arms and slow significant looks.* Had we not some friendly regard to the memory of the Doctor, we should stop to make some remarks on these portentous *arms and looks*—but let us read the whole fray as related in the book p. 243, after 54 years of sleeping and waking dreams.

"In my Inaugural Dissertation, as first printed, I inserted a brief account of my letter from Lancaster, respecting the cure of fever by a shower of rain, and the purpose to which Dr. Rush had applied it. That account, however, I had expunged, at the suggestion of the Dean of the Faculty, who frankly told me that he knew it to be unnecessary and useless, and deemed it improper. Still, during my defence of my thesis, Dr. Rush, who was my objector and opponent, referred to it with great virulence and blame. In relation to that objection, however, which was the first he introduced, I completely overthrew and silenced him, and gained by that means a decided advantage in everything that followed.

No sooner had the doctor emptied on my letter his first vial of wrath than I rose, and, addressing the Rev. Dr. Ewing, the Provost of the University, who presided on the occasion, said with great calmness, and in a suppressed tone, 'I was summoned here, sir, as I was given to understand, and of course to believe' (laying on 'believe' a strong emphasis) 'to defend only what is contained in my thesis; not what I have stricken out of it. But if it be your decision (emphasizing 'your') that I shall defend also the expunged passage, I am perfectly prepared for the task, and will cheerfully perform it.'

'It is not my decision that you should defend the expunged passage,' said the provost, in a very decisive tone; 'and Dr. Rush has no right to refer to it. In doing so, he is out of order.'

I bowed and resumed my seat, persuaded that a sparring match between the professor and the provost would immediately ensue; for they had never played towards each other the brotherly parts of Orestes and Pylades.

Dr. Rush positively and vehemently declared that he had a 'right to refer to the letter,' and to call on me to defend the account of it which I had inserted in my thesis; and that he would maintain that right. The provost then, addressing himself to me, said: 'Did you not say, sir, that you had expunged from your dissertation the passage in which the letter is mentioned?'

'I did, sir; and I said so truly. The passage was expunged by me, at the suggestion of the Dean of the Faculty, and is not now in my thesis.'

'I hold in my hand,' replied Dr. Rush, 'a copy of the thesis, in which, at page —, the passage still remains.' In a moment the trustees, to each of whom two copies of the dissertation had just been given, turned to the page mentioned by Dr. Rush, and unanimously asserted that the copies of the pamphlet which they had just received contained no such passage.

I then approached Dr. Rush, with a hurried step, and said to him abruptly, and I doubt not half-mandatorily: 'Pray, sir, allow me to see this pamphlet!'

'Do you doubt the authority of my word, sir,' said he, in an indignant tone, 'as to the contents of your pamphlet, and therefore demand a sight of it yourself?'

In a voice no less indignant, I promptly replied: 'In the present case, sir, as respects the assertion you have made, I doubt all authority but my own eyes;' unceremoniously taking the pamphlet out of his hand.

'Then use your eyes, sir, to your own *conviction*, and the verification of my word,' was the professor's terse and stern rejoinder.

'I have used them, sir, to my full conviction.'

Turning then to the provost, with the pamphlet raised aloft in my hand, so that every one in the hall might see it, I added, in a tone of cutting sarcasm: 'This is a spurious copy of my thesis, procured by what device I know not, and brought here for what purpose I care not.'

Turning again to Dr. Rush, I continued in the same tone: 'You must know this, sir, to be a counterfeit copy of my thesis; for I can prove your having been apprized yesterday that the passage you except to was erased by my direction, at the suggestion, as already stated, of the Dean of the Medical Faculty.'

And I then disdainfully tossed the pamphlet on the table before him, and returned to my place. But, instead of sitting down, I maintained my standing position, drawn up to my full height, with folded arms, and looked slowly and significantly towards that portion of the audience formed by the Trustees of the University and the Faculty of Medicine.

For a moment, not a word was spoken, except in whispers. But, at length Dr. Rush, agitated by a mixture of passions, mortification and resentment being the leading ones, said to me in a half suffocated voice: 'By whom, sir, do you presume to assert that I was informed of the offensive passage being stricken out of this pamphlet?' the thesis being again almost spasmodically grasped in his hand.

'I have said nothing about that pamphlet in your hand, sir, except that it is a counterfeit. But I say that Mr. D—b—n, the printer of my thesis, informed you yesterday that the passage so often referred to was erased by my order. And if he be in this assembly, he will testify to the truth of my assertion.'

'Sir, Mr. D—b—n did not tell me that the passage was stricken out; but only that it was to be stricken out. But finding it still here,

(pointing to the pamphlet), 'I feel authorized to suppose the order to be withdrawn.'

'Sir,' I replied, with increased indignation, 'the pamphlet now in your hand is the same that you possessed when you saw Mr. D—b—n, before he had made the intended erasure. Had you looked into the copy which, by my direction, he sent to you this morning, and which, I doubt not, is in your possession, you would have perceived that my order to him had been faithfully executed. And there would then have been neither plea nor excuse for this altercation, on account of which I am both mortified and ashamed; yet, for the production of which, I appeal to the audience that I am not in fault—for it is exclusively the product of your own groundless and unjustifiable resentment.'

It was now, that by his vehemence, Dr. Rush drew from me the haughty reply which has never since been forgotten; yet, as far as I know, never altogether correctly reported. It soon after its occurrence, found its way into two or three of the Philadelphia newspapers, and has since been several times republished in New York and Boston papers, and perhaps in those of other parts of the country.

Almost hysterical with rage, the doctor said to me, immediately after the utterance of my last sentence: 'Sir, do you know either who *I* am or who *you* are yourself, when you presume thus arrogantly to address me?'

'Know you, sir?' I calmly, but contemptuously replied. 'O! no; that is impossible. But, as respects myself, I was, this morning, Charles Caldwell; but indignant, as I now am, at *your injustice,* call me if you please, *Julius Cæsar, or one of his descendants!*'"

If such a scene as this occurred in the University, it was high time to turn the trustees and professors headlong out of the dishonoured temple. Would the respectable strangers, the Bench, the Bar, &c. who were present, send their sons to a college thus misgoverned? But the truth must be, that no such thing was ever seen there as here related, though Dr. C. says that part of it was published in Philadelphia newspapers "soon after its occurrence." Peter Porcupine *alias* William Cobbett was then a very notable busybody in Philadelphia, and assailing Dr. Rush the next year with all his energy and venom, he may have caricatured whatever occurred at Caldwell's doctoration, in the Gazette which he began about this time. But conjectures though plausible are vain and researches even if successful would prove nothing; for C. says that the part published was never, as far as he knows, "altogether correctly reported."

But Dr. Rush is the second hero of this romance. He was the chief of those adverse spirits of whom our author says,

"But for them I should have been a professor in the University of Pennsylvania, occupying the chair then filled by Dr. Rush, and now by Dr. Chapman, but created by their predecessors, instead of being at present a professor in the University of Louisville, occupying a chair created by myself. Which of the two situations would have been most profitable to me, and which most toilsome and hazardous, are questions easily solved."

Now remember that he had set his magnanimous heart on this very chair when he first heard Rush's eloquence therein, p. 49; and that he never ceased to labour hard therefor, till he found that Dr. Chapman had, by "his superior tact," anticipated the favour of the trustees, p. 50. This failure of the long hope, was the chronic irritation that left him in a fretful state all the rest of his life; this no doubt was the subject of many of his waking and even his sleeping dreams, until these had jostled realities out of his mind and made him describe things as they never existed and relate speeches that were never made. Doctor Rush used to tell us of a gentleman who wisely forbad his children to relate their dreams, lest they might sometimes confound them with realities, and thus lose their regard for truth. *Sunt geminæ somni portæ*, says Virgil.

> "Two gates the silent house of Sleep adorn,
> Of polished ivory this, that of transparent horn;
> True visions thro' transparent horn arise
> Thro' polished ivory pass deluding lies."

Through this delusive gate came the *dream* related in p. 206–7–8. He tells circumstantially how he saved the lives of Mrs. Gen. Gurney and her daughter in the year 1794. The lady must have been almost frightened to death and the Doctor anhelitous with overpowering exertion, yet they both make eloquent speeches; nor could the author have made them better in his 80th year—nay, I presume they were just such as he would then have written, breathing gently at his table and in the maturity of his intellect. But true history coming through the gate "of transparent horn," relates that Gen. Gurney had no children by his first wife, that he married his second wife during the yellow fever of 1793, and that his oldest daughter, who the Doctor says was 9 or 10 years old when he saved her life in 1794, was not born until the latter end of the year 1795. Tis vain to say that some little *friend* was mistaken for a *daughter*, for Dr. C. says, that the acquaintance, thus begun, "ripened into friendship uninterrupted to the end of Gen. Gurney's life." Two daughters of the General now live in Philadelphia of sound mind; they acknowledge the friendship of their father for Dr. C. but they never heard of any even the least saving effort made by the Doctor, till they read it in his Autobiography.

This *cacoethes* of speech-making is the greatest blemish in the workmanship of the book, for it gives to the whole mass the character of romance. It is an offence that is often met with among those troublesome mortals who are indecorously ambitious of eminence in conversation, without the ability of either instructing or amusing. Some men fond of relating their victories and their superlative shinings in company, will grieve you with the relation of a long harangue which you are certain was never made; and yet for civility's sake, you are obliged to listen to, assent to, and thus to applaud the flowing oration. We knew a lawyer who was thought to practice oratory on every company; an excellent lady was asked—had you a pleasant time last night; no, Mr. H. practised upon us, but we had no conversation.

During the yellow fever of 1797, our hero and Dr. Rush had a reconciliation, the cause of which does honour to them both. Rush was strenuously pursuing his treatment of the fever, in which he was followed by Barton, Physick, Caldwell, Griffitts, Currie, and others; but the persecution of the opposing party with Porcupine at their head, fell upon Rush alone, as says Horace, "thunderbolts strike the tops of the mountains." Dr. C. says p. 278,

"For several reasons, but principally for *two*, this state of things soon became to me intolerable. In the first place, Dr. Rush, as I believed then, and still believe, was contending for the right, in both practice and theory; by which latter term I allude to his doctrine of domestic origin. To defeat and injure him, therefore, would be tantamount to an injury to the community at large. And in the second place, let the issue be what it might, and the principle involved be what it might, to witness a contest of dozens against one, without some sort of interference in behalf of the oppressed, was an act cold-hearted, illiberal, and unmanly, from which my nature recoiled, with sentiments akin to contempt and abhorrence. True, Dr. Rush was not my friend. But no matter for that. In the present case he was much more. He was the friend of truth, and of his race, and deserved to be supported by every man of honor and virtue. Besides, I had long since determined never to allow private feeling to impede the discharge of public duty."

Dr. C. then wrote anonymously in defence of the new doctrines and, p. 279 "drew the current of the daily slanderst hrough the press, very much from Dr. R. thus giving him a little respite from the galling annoyance he experienced." Rush heard that C. was seized with the fever and discovered at the same time that he was the author of the defensive papers. He sent immediately to Caldwell and requested that himself and Dr. Physick might be admitted as visitors. They were kindly received and they attended him through his disease. C. says, p. 281, "R. exercised towards me the whole resources of his amenity and courtesy which were almost boundless, for he was among the most polished men of that polished age." Now even here, most wonderful to tell—these great men were not permitted to retire for consultation till their patient, very ill of yellow fever, had made them a long speech to convince them, as he says, that he knew something of his own case and that he had no fear of death!

Having gone through the epidemic of 1797, he gratulated himself in these words, p. 283—

"I had extended materially the sphere of my business, improved my reputation as a writer and practitioner, and, as far as appearances were concerned, and with as much reality as I ever expected, had smothered my misunderstanding with Dr. Rush—and I had done so in a manner triumphantly in my favor."

He now pursued his studies with his usual vigor, determined to leave no stone unmoved in preparing himself for a professorship, for a man he says, p. 287,

"Destitute of medical literature and science, and undisciplined in composition, reading, and speaking, seated in the chair of a medical professor, constitutes one of the fittest of 'objects for scorn to point her slow unmoving finger at,' and for all well qualified and high minded teachers to treat with contempt."

He therefore spends ten more laborious years in fitting himself for public instruction. During this time he translated *Senac* at the request of Dr. Rush, to whom he dedicated it in the most affectionate language and sentiment. But the dedication was made to R. without his knowledge, because his modesty was so enormous that he would never have permitted it; and it was made to him says Dr. C., because he was among the ablest judges in the whole world of the merits of the work.

"But I am actuated, also, by other considerations, which though more private and personal in their nature, are not with me less powerful in their operation. These considerations, were they to be even rejected by the judgment, would appeal to the feelings, and though repulsed from the head, could never fail to gain admission to the heart.

During an intercourse of some continuance, particularly during the term of my medical pupilage, and the first years of my practice as a physician, I received from you many acts of attention and courtesy, which as a young man and a stranger in the place, impressed me deeply at the time, and have still continued to be sources of grateful recollection. Out of these civilities, obligations naturally arose on my part, which our relative situation has not yet allowed me to cancel. It is even possible, that an opportunity of cancelling them may never occur. I must, therefore, beg your acceptance of this dedication as some acknowledgement of them, accompanied by my sincere wishes for a long continuance of your health, happiness, and *useful labors.* For however grateful, in the evening of life, the *otium in secessu honestum* may be to a philosophical and contemplative mind, I am unable to wish you such a retirement. It is enough that we should be deprived of your labors and services when you shall have gone to enjoy the reward of them in a better world. CH. CALDWELL."

Truly this would seem to show that the old sores were quite healed, and even that the original cuts had not been so deep and ghastly, as they are now represented. This affectionate dedication of Senac is dated Oct. 1805, and only 15 months afterwards, he published an excellent history of the yellow fever of 1805 wherein he applauds the practice he learned from his old Master, but does not once name him, however renowned in these epidemics. It was during these 15 months we presume, that the conversation took place related in p. 289 which we shall quote entire. Speaking of his preparation for a professorship, he says—

" It was uninterruptedly and laboriously continued during the space of ten years. True, within that space I delivered to pupils, by invitation, many addresses on medical subjects. But ten entire years had elapsed, before I ventured to call together a class to listen to my lectures for the purpose of instruction.'

'Though, in my Philadelphia lectures, I broached many sentiments, and presented and defended many views in direct opposition to those entertained and inculcated by Dr. Rush; yet, in all cases, I spoke at first of his opinions so courteously and respectfully, and of himself so complimentarily, that it was hardly possible for him to except to anything I uttered. Yet did I plainly perceive that he was not satisfied with the self-resource doctrines I irrespectively taught, and the independent course I pursued in relation to them. Nor could I fail to be made sensible that our intercourse became less and less cordial. Still, however, did the doctor occasionally refer to the subject in such a way, as to present to me the prospect that, in case of certain contingencies, the doors of the Medical School of Philadelphia would be opened for my admission to a professorship. But even on that subject, his encouragement grew fainter as time and changes went on; until he at length gave me to understand his opinion, if not his wish to be, that, except on certain conditions performed on my part, the doors of the school would be certainly closed against me; and with those conditions he well knew I would never comply.'

'Pray, sir,' said I, 'have the goodness to inform me whence has arisen this sudden change?' He replied that the change was not very sudden, but had been in progress for some time. 'Wherefore, then,' I rejoined, in an excited tone and manner, somewhat resembling those of *demand*, 'have I not been apprised of it at an earlier period?' 'Why, sir,' said he, 'to be the announcer of unpleasant news is an unpleasant employment.' 'It is, or surely ought to be,' I promptly replied, 'less unpleasant, and more friendly and useful, to communicate the news of things being in jeopardy, but still perhaps remediable, than of their being lost and irremediable.'

'But whether the change referred to be of recent or of long standing, it has a cause; and of that I hold myself entitled to be informed.' 'Though I am not,' said he, 'in the habit of divulging the existence of secret and alienated feelings, it is perhaps my duty, in the present case, so far to do so, as to tell you, that some members of the Faculty are not friendly to you, and are unwilling to speak well of you to the Board of Trustees.'

'Unwilling to speak well of me! Do any member or members of the Faculty dare to speak ill of me to either the Board of Trustees, or any other persons? If so, I have a right to their names.' 'Of your talents, attainments, and powers in lecturing and instructing,' he replied, 'they speak in the most respectful and flattering terms. But they are reluctant to recommend you to the Board of Trustees, in the light of a professor.'

'It is time enough for them, sir, to refuse their recommendation, when it is wanted. I have never either asked for it, or coveted it. Nor do I set on it the value of 'a pin's point.' And you are authorized, if you please, to tell them so, and say that you do it at my request. The only recommendation I rely on, or would accept, 'is that of my fitness to discharge the duties of the station. And that 'fitness' not a member of the Faculty ever has denied, or will deny, in either my own presence, or in that of my friends. And you may deem it even superfluous in me to add, that to yourself the truth of this is thoroughly known, and by yourself has been publicly and repeatedly acknowledged. And to you is it further known that, had I, like two or three other persons, whom it would be superfluous in me to name, degraded myself and flattered them, by paying court to certain members of the Faculty, who need not be designated to you, it would have been very easy for me to conciliate their patronage and favor, and procure their recommendation to any appointment I might solicit or desire. For the only reason they have to decline speaking *well* of me to the Board of Trustees, is because I have never condescended to speak flatteringly of them.

'But this conference is no better than a waste of words. I shall therefore close it by remarking that, though you have pronounced the Philadelphia Faculty barred against my entrance, either it or some other will yet be open to me, and I shall be invited and solicited to enter it. For, notwithstanding the hostility towards me, to which you have alluded, should my life and health be spared, I will, before the lapse of many years, be the occupant of a chair in a school of medicine as honorable as your own.' This was the last conference I ever held with Dr. Rush. And though, for some time afterward, we civilly saluted when we met, we at length discontinued even that mark of regard, and passed each other without recognition."

His hopes, as we learn from his book, were that R. would give him the *Institutes*, confining himself to the *Practice;* and the book proves that the aspiring man was rather more obtrusive and demanding than modesty permits. Rush, moreover, could not be expected to bring forward a man who had been lecturing against his doctrines. True this very thing was done many years afterwards and in that very chair; but this was in circumstances so different, that the two cases cannot be brought into parallel.

It was in the year 1809, we believe, that C. began to interrupt Rush's lectures. He rented a room in Market st. above 9th and advertised the students of his lectures by placing his servant at the gate of the University with bills. His first two or three lectures were pretty well frequented, but the number rapidly declined. I was persuaded one luckless evening to hear him in the 2nd st. market house whither he had removed; and had I not gone, it would have been another case of the *Dearly beloved Roger, you and I,* for William W. Anderson of Maryland and myself were the whole class—*nos duo turba sumus.* We were told that timely notice would be given of the next lecture, but none reached us till November of the next year, when something like the same farce was played and with the same final success. Whether anything was attempted in subsequent years, we do not remember.

He had now no hope from the University during Rush's life, and therefore he had the less pain in following his old master to the grave in Ap. 1813. On this occasion, he seems to have seen things, not as they were but as he wished them to be, for he tries to persuade his readers that the death of the Old Man, as the Greeks used to say, made very little sensation in the city or any other place. This is another proof that his dreams came out at the "ivory gate," for it is in direct opposition to all that we have ever heard, not having been present ourselves. "No member of the Profession of any standing either volunteered his services or could even be induced to pronounce on him a eulogy," p. 314. This language conveys a falsehood—that one or more were desired to do this and spurned the office. But the truth is, that no physician in Phila. at that time could have felt himself adequate to the task, except Barton, Caldwell, and Chapman, who were all personal enemies of the defunct, and therefore, and for other rersons, could not have been agreeable to the bereaved family. But there was "no flood of tears from the eyes of the citizens generally or even those of his own immediate neighborhood," p. 313. Truly many a man has deep sorrow at his heart who neither sheds tears nor makes a wry face. This melting Doctor seems to require too much of us common mortals; he looked for streaming eyes, having said that Senac was followed to the grave "by thousands in tears."* Well may we exclaim with the Satirist—*Mirandum est unde ille oculis suffecerit humor.*

But Dr. C. is not always consistent with himself. In his life of Rush *in Delaplaine's Repos.* he says—"since the death of Washington, no man perhaps in America, was better known, more sincerely beloved, or held in higher esteem. Even in England the *tear* of sensibility descended on his ashes and the voice of eulogy was raised to his memory." * * * * "For nearly 3000 years past, but few physicians equal in greatness have appeared in the world; nor is it probable that the number will be materially increased for ages to come." *Si sic omnia dixisset,* for in the book before us, he labors hard to depreciate the medical, philosophical, and social character of his master. But all who knew the sterling worth of this great and good man, will look upon him, though willing to be called a *descendant of Julius Cæsar*, as the ass kicking the dead lion.

He says p. 314, that all Rush's theories died with him and that some of them he even survived. This is not true, as every

* Caldwell's Preface to Senac.

one knows; but true it certainly is, that at his death, they fell "on evil days and evil tongues." Rush was succeeded by Barton who had been his sworn personal enemy for many years and had opposed him in everything except his doctrines and treatment of yellow fever. This man now published an edition of Cullen with some notes and made it his text book; he revived Nosology, and sought every means of depressing his great predecessor.

This most unhappy man sank to rest very quickly under his new labors, when forthwith came Chapman, another bitter personal enemy to Rush. He labored assiduously to undermine the reputation of his great Preceptor; and in this work, he was cheerfully assisted by Caldwell who secretly aided him in preparing his lectures. Barton had introduced Cullen's *F. Lines*, and therefore Chapman now prevailed on Caldwell to publish a new edition of this work with voluminous notes. This book, our new professor used for twelve years.

Meanwhile the two Friends patched up a lame and stumbling theory which they dignified by the word *Sympathetic.* In this theory, says Caldwell in his letter to Chapman, *Ph. Med. and Phy. Jour. vol. iii.* 303, "it is certainly true that as teachers and disputants, we have for many years held our station in the first rank of battle." Lame as this doctrine was, it stalked abroad, no doubt like Sir John Mandeville's nation of Ethiopians who had but one leg; for we take it for granted, that Sir John exaggerates when he says, they could run faster with only one leg than other nations can run with two.

Meanwhile the Lectures of Rush were not published by his family, but retained to be delivered annually by his son, with such addition and subtraction, perhaps, as he might think proper; by which suppression, the long, and careful, and anxious experience of this profound medical philosopher has been lost to the world and to his own fame.

That Dr. C. was a man of superior natural mind made powerful and commanding by unwearied labor, there cannot be a question. His early education, he acquired on hard terms and not without great and meritorious exertions. Being thrown early in life on the resources of his own mind, his early learning was principally that which use required, hence he became a translator from Latin and French; but we do not see or hear that he made much progress in Greek and other academical studies, except in one of his conversations with Dr. Rush. In his *Reply to Haygarth*, which he seems willing to suppose sent the Doctor "where the wicked cease from troubling," he makes a host of blunders in the use of a *regular* Greek adjective. Ridiculing him for the phrase "*gratefully thankful*," Caldwell exclaims—"in plain English *thankfully thankful!* How brilliant is the commencement of the 19th century! a new degree of comparison ushered into the world to supply the deficiency of the former three! The French have only their *reconnaissant*, *plus reconnaissant*, *bien reconnaissant;* the Romans only their *gratus*, *gratior*, *gratissimus;* the Greeks only their *eucharis*, *euchariôn*, *eucharistos;* but thanks be to the genius of Haygarth, we have our *thankful*, *more thankful*, *most thankful*, and *thankfully thankful*." This, it must be confessed, is rather petulant than wise; ignorance, it certainly is. Eucharis is not compared as he has done it, but regularly in *teros* and *tatos*. There is no *eucharion* in the language nor *eucharistos* in the superlative degree. Finding *kalos*, *kalliôn*, *kallistos* and *kakos*, *kakiôn*, *kakistos*, he compared *eucharis* in the same way! Had Dr. Haygarth replied, he might have shamed him for his impudent ignorance.

In early life he appears to have been an imitator of Johnson and Gibbon, as seen in his dedication and version of Blumenbach; even in later life, his early grandiloquence sometimes escaped him. He says of Rush, as above quoted—"even in Europe, the tear of sensibility descended on his ashes and the voice of eulogy was raised to his memory." Here he looked to the roundness of his period rather than to the sense, for eulogies naturally rise and tears fall—though not from Europe on our *ashes* in America. When Editor of the Port Folio, he requested his contributors to send him "something equally remote from intangible levity and insupportable onerosity." But this *onerous* and *insupportable* diction is not often seen in his later works; these afford a strong masculine style, natural and perspicuous, free from affectation, and not disgraced by words of his own coining.

His whole character, like his manner of writing, was formed by intense labor and care; whatever he did, he always tried to do in the best manner, hence he was in early life, like his early style, rather an unnatural man, the result of perpetual affectation. Thus I can remember telling a lady about the year 1807, that if he scraped his feet at the door, he would try to do it with the utmost grace and dignity. This opinion of 48 years ago, accords with one of his three rules of conduct adopted by him when yet a boy—"whatever you do, do as well as you can." p. 443. But when he visited Philadelphia, I believe in 1839, this extreme politeness seemed to have worn itself into a more natural grace and easy dignity.

One of the greatest faults of his life is, that he wrote on too many subjects, and of this he was finally convinced. Had he devoted his mind to his profession, he might have left some lasting monument to his name and country; but the dilution of his powerful intellect has been such, that it may be a question, whether any of its numerous progeny is strong enough to survive.

If we consider his entire history, he must be looked upon as an extraordinary man. He was not brilliant but learned; his acquisitive faculties were strong, nor in ratiocination was he at all defective; but imagination was wanting, without which no man can ever become truly great. He deserves much praise for his industry and his uninterrupted temperance; and yet even these virtues may be suspected of selfishness, for his ambition was so inordinate, that he seems to have had the *sublimi feriam vertice sidera* of Horace perpetually in his mind.

Had he not ambitiously overstrained some of his virtues, he would have been a more agreeable, popular, and useful man; had he been less obtrusive, fame would have followed him as it was said to follow the modest Cato; and had he been decently respectful to his old master, to whom by his own confession he was greatly indebted, he would have been the successor of Rush and have proved a lasting honor to American Medicine: but *Omnibus in terris quæ sunt &c.*

> "Look round the habitable world—how few
> Know their own good, or knowing it pursue."

*A treatise on the Practice of Medicine.* By George B. Wood, M.D. Professor of the Theory and Practice of Medicine in the University of Pennsylvania; President of the College of Physicians of Philadelphia, etc. Fourth edition. Philada.: Lippincott, Grambo & Co. 1855.

When the above publication first appeared in 1847, many excellent treatises on the practice of medicine were in use throughout our country. Notwithstanding these, it took its position at once as a standard work of the very first order. The distinguished reputation of its author, as one of the writers of the United States Dispensatory, a work which is universally acknowledged to have no equal of its kind in any language, as well as the fact that he was extensively and favorably known as a teacher in the first school of our country, contributed somewhat, undoubtedly, to this brilliant success. The exhaustion of three large editions in eight years is a sufficient and convincing proof, however, if any such were needed, that the high character at once accorded it by the profession did not owe much to such extrinsic causes, but was due to its own inherent and indisputable merit. A sound and well disciplined understanding brought to bear upon subjects with which the writer was familiar, both from his own personal observation and experience, and from intimate acquaintance with and study of the writings of others, was evidenced throughout its pages. At this late date, commendation of it, however, is useless. We shall only say, therefore, that as a clear, exact and truthful representation of the science of which it treats, we think it will bear a most favorable comparison with any of the systematic treatises that have appeared in our language.

The progress of medical science at the present time is so rapid, the sources from which the new facts and observations of writers must be gathered are so numerous, that frequent revisions and alterations are required to be made in every standard treatise, if its author wishes to keep apace with the ever flowing current. To make these necessary changes, demands a great deal of labor and much discrimination. A vast amount of chaff must be winnowed before the bright grains of truth can be gathered. The present edition bears evidence throughout its pages that these duties have been well executed by our author. Besides the numerous alterations in the text, and the many additions, principally in the form of notes, which are given us, we notice that some of the sections have been considerably enlarged and modified, as, for instance, the one which treats of "Disease of the fluids." We need hardly say, that the result has materially enhanced the value of the work.

Upon the subject of peritonitis from intestinal perforation, occurring during the course of typhoid fever, we observe a note in the present edition, (see p. 349,) which states that the author had successfully treated another case of this grave accident. "It must be confessed, however," he remarks, "that the value of such observations is diminished by the publication of certain cases by Dr. Thirial, which go to prove that violent and fatal peritonitis, which might readily be mistaken for peritonitis from perforation, occasionally occurs in enteric fever without any discoverable opening in the bowel." (See Med. Examiner, N. S. xi. 120.) As the subject is one which may prove interesting to some of our readers, we shall make a few comments upon it.

Though perforation is occasionally mentioned by some of the early writers, M. Louis may be said to be the first who drew the especial attention of pathologists to its frequent occurrence in typhoid fever. In his *Recherches Anatomiques, Pathologiques Ete de Fievre Typhoïde*, he states that he had found it to exist in eight cases out of fifty-five examined by him, or in nearly one seventh of the fatal cases. Ordinarily there is but one perforation, which generally takes place in the neighborhood of the cœcum. In five of these eight cases, the previous fever was either slight or latent. In one instance, it occurred on the 12th day of the fever; in two on the 18th; in the other four, between the 22d and 42d day. Severe symptoms marked its occurrence in five cases. In the other three, it was latent, so to speak, although death took place quite as rapidly as in the others. The symptoms were sharp and tearing pains suddenly experienced in the abdomen, rapidly followed by change of expression, nausea and vomiting, rigors and all the characteristic marks of an intense acute peritonitis. "We can consequently," he says, "diagnosticate that a perforation exists, whenever the following characteristic symptoms occur. If in the course of a typhoid fever, grave or slight, or even where unexpectedly, the disease having been latent until such occurrence, severe pains in the abdomen occur suddenly, which are exasperated by pressure, accompanied with alteration of the features, and, sooner or later, by nausea and vomiting, we may announce that there exists a perforation of the intestine." Each of the above symptoms, he remarks, however, must be present to enable us to form a positive opinion. Not only must the pain be violent, with marked alteration of the physiognomy, it must also be increased by pressure, and sooner or later extend over the whole abdomen. Even though great diminution of the pain should occur, we should still adhere to our previous diagnosis, should all the other symptoms still persist.

Our knowledge of the symptoms characterising peritonitis from perforation, has received no further increase since the date of the above generalization. The formula may still be considered good, though as we shall hereafter show, there are occasional exceptions to the universal adoption of it as a law.

The prognosis of perforation, it is almost needless to observe, is most disheartening. In fact, until within a few years, its occurrence was considered as invariably fatal. Dr. Stokes, in an article contributed to the Dublin Medical and Chemical Journal, was the first to cast a doubt upon this. After showing the inapplicability of the previous methods of treating it by blood-letting, mercury, &c., he observes that the two great indications are to support the strength of the patient so as to gain time, and to diminish, as far as possible, the peristaltic action of the intestines. If this latter object can be attained, not only might the further ingress of fœcal matter into the peritoneal sac be prevented, but what is equally important, the organizable lymph effused would be allowed to perform its office undisturbed. To attain these importants ends, he proposed the use of opium in large doses, and detailed two cases in which " decided evidence of the utility of this mode of treatment was obtained. In the first, in which the symptoms of perforation had existed for two days, and the patient was in almost complete collapse, the use of sixty drops of black drop in the twenty-four hours was followed by most singular improvement." In forty-eight hours, " *every symptom of abdominal inflammation had subsided;* the belly was natural and the pulse good. At this period the mildest possible laxative was exhibited, which produced four evacuations, followed by an immediate return of the symptoms of peritonitis, under which the patient rapidly sank. On dissection, we found universal peritonitis, but the intestines were everywhere agglutinated together, except in the left iliac fossa. The perforation existed in the cœcum and was small; and the mucous membrane of the ileum and color was but little affected."—(*Cyclopedia of Prac. Med. Art. Peritonitis*, p. 528, Am. edition.)

"In the next case, the disease was of three days standing, and it supervened suddenly from a hypercatharsis, produced by an over dose of Glauber's salts. The patient was apparently in the last stage when the opium treatment was commenced." Under its use the symptoms soon improved, and at the end of ten days, after taking 105 grains of opium, convalescence was completely established.

Since the period of publication of Dr. Stokes' article, several cases of supposed cures of perforation have been published. In all of them, opium or some of its preparations, was freely administered. Two of these presumed cures, both of which are referred to in the present edition, have been published by Dr. Wood. The first took place in 1850, the particulars of which, as stated to the College of Physicians of Philadelphia, may be seen in its Transactions, vol. 1, p. 32, N. S. The second one, which occurred in the latter part of the year 1852, though of a less certain character than the first, presented features which rendered it "highly probable that the inflammation of the peritoneum resulted from perforation of the bowels."—*Ibid*, vol. 1, p. 418, N. S.)

Such was the belief entertained by the profession, regarding the possible cure of this affection, when Dr. Thirial published an article in *L'Union Médicale*, Nos. 83, 84 et 85 ; 1853, in which he detailed four cases of peritonitis which occurred in the hospital *de la Charité*, during the service of M. Rayer. One of these, was admitted for favus ; two for typhoid fever and one for disease of the heart, which was followed, three weeks after admission, by an attack of typhoid fever. In the three latter cases, the patients were severally convalescing from their diseases when symptoms of acute peritonitis, with rapid sinking of the vital powers, attributed in each case by the attending physician to the occurrence of perforation, suddenly supervened. Death rapidly followed in all of them. The autopsies, which were made with the most scrupulous care, were conducted in the following manner. The intestines were detached and then filled water, the precaution being first taken of tying a ligature around the lower portion. No solution of continuity being discovered in them by this method, they were then laid open and minutely examined. Still no perforation could be found. Vivid injection of the whole peritoneum, soft and recent plastic lymph, serum, false membranes, in fine, all the pathological evidences of acute peritonitis were alone revealed by the examination. The diagnosis of perforation was found incorrect in every instance.

In the present number, we record two additional communications by Dr. Wood upon the same subject. To these, and especially to his remarks on the second case, we invite the attention of our readers. Difficult as it must be to arrive at a positive determination upon the point at issue, viz., whether a perforation has *ever* been cured, we think our readers will agree with us in considering it as very improbable, to say the least. When we reflect that acute peritonitis, even in a previously healthy person, is a very dangerous affection—that our prognosis would naturally be still more unfavorable, should it suddenly occur either during or immediately after, an attack of typoid fever ; its conjunction under such circumstances, with such a fearful accident as perforation, must, *a fortiori*, leave hardly a doubt of the result. Add to these considerations, that perforation has no symptoms, *per se*, separate from those which belong to acute peritonitis, by which we can detect its presence—that it has been fairly proved, also, that peritonitis does occasionally occur, both during and after typhoid fever, with no perforation, and we think it will be difficult to evade the conclusion that in every instance where recovery has been supposed to have taken place, uncomplicated peritonitis has been the disease under treatment.

*Essays on Infant Therapeutics: to which are added observations on Ergot; history of the origin of the use of Mercury in Inflammatory complaints, &c.* By JOHN B. BECK, M. D., Professor of Materia Medica and Medical Jurisprudence in the College of Physicians and Surgeons of the University of the State of New York, &c. Second Edition, enlarged and revised. New York, S. S. and W. Wood, 1855.

The above is an able and valuable work, abounding in much useful information, from which all may reap benefit, but more especially adapted to the use of the young practitioner. The research, sound judgment, and good sense, for which its lamented author was so highly distinguished, are most effectively portrayed in it. The practical nature of most of the essays, treating as they do of the evils resulting from the excessive use of remedies daily employed by physicians, claims for them particular attention.

